# Revisiting the Role of Mitochondrial DNA Mutations in Aging: Bridging Theoretical Expectations with Empirical Observations

**DOI:** 10.14336/AD.2024.1469

**Published:** 2026-01-31

**Authors:** Runyu Liang, Qiang Tang, Jia Chen, Yongyin Huang, Luwen Zhu

**Affiliations:** ^1^Heilongjiang University of Chinese Medicine, Harbin, China.; ^2^The Second Affiliated Hospital of Heilongjiang University of Chinese Medicine, Harbin, China.

**Keywords:** Mitochondria, Mitochondria DNA, Aging, Senescence

## Abstract

Since the association between mitochondria and aging was first identified, significant efforts have been devoted to elucidating the role of mitochondrial DNA mutations in the aging process. Due to their age-dependent accumulation, intrinsically high mutation rates, and defective replication mechanisms, mtDNA mutations have often been regarded as pivotal drivers of aging. This has led to certain intuitive yet inherently limited conclusions. Aging, however, is a multifactorial process, and the role of mtDNA cannot be simply categorized in binary terms, as its influence emerges as a composite vector of numerous interconnected physiological processes. Adopting alternative perspectives may mitigate the discrepancies between theoretical expectations and empirical findings, offering new directions and insights for future research.

## Introduction

Mitochondria, originating from ancient symbiotic bacteria and thought to be descendants of α-proteobacteria, provide most eukaryotic cells with remarkable energy-generating capacity [1, 2]. This unique organelle is not only central to cellular metabolism but also harbors a distinct circular genome, known as mitochondrial DNA (mtDNA), whose replication and regulation are independent of the nuclear genome. The mtDNA encodes 13 essential proteins involved in oxidative phosphorylation (OXPHOS), a process that generates approximately 90% of cellular bioenergy by oxidizing hydrogen derived from nutrients and combining it with oxygen [3, 4]. Beyond energy metabolism, mitochondria play pivotal roles in calcium homeostasis, iron signaling, steroid synthesis, reactive oxygen species (ROS) generation, and programmed cell death. They also serve as critical nodes of epigenetic regulation, profoundly influencing gene expression and cellular function [5-7]. Albeit, the indispensable role of mitochondria in biology is counterbalanced by their vulnerability to dysfunction under intrinsic and extrinsic stressors. Their genetic material, mtDNA, is highly prone to mutations, which are widely regarded as key drivers of various diseases due to their elevated mutation rate [8].

Mitochondrial diseases are typically heterogeneous and multisystemic, characterized by impaired energy metabolism, lactic acid accumulation, and other symptoms. Tissues with high energy demands, such as the brain, muscles, heart, and endocrine system, are particularly susceptible to energy deficits caused by hereditary mtDNA mutations [9, 10]. Classical mitochondrial diseases, including Leber’s hereditary optic neuropathy (LHON) and mitochondrial encephalomyopathy with lactic acidosis and stroke-like episodes (MELAS), rank among the most common inherited metabolic disorders, with an estimated incidence of one in several thousand newborns [11-13]. Yet, the impact of mitochondrial dysfunction extends beyond genetic disorders. In recent years, mtDNA mutations and mitochondrial dysfunction have been implicated in a wide array of common human metabolic diseases, including diabetes and obesity, which often arise from the progressive decline of mitochondrial function [14, 15]. Over longer timescales, these defects contribute to cellular aging and the onset of age-related diseases, such as Alzheimer’s disease, Parkinson’s disease, cardiovascular disorders, and cancer [16-19]. Thus, mtDNA mutations play a central role in the biological continuum from genetic disorders to chronic metabolic conditions and the aging process.

Compared to nuclear DNA(nDNA), mtDNA is inherently more susceptible to mutations due to several factors: its replication is independent of the cell cycle, it lacks the protective mechanisms of nDNA repair systems, and it is exposed to a high level of oxidative stress [8, 20]. The mechanisms underlying mtDNA mutations are complex and multifaceted, involving replication errors, oxidative damage, environmental stressors, and the unique selective inheritance of mitochondria. These mutations can manifest as point mutations, deletions, or rearrangements, ultimately compromising OXPHOS function, disrupting mitochondrial bioenergetics, and causing widespread cellular dysfunction [21-23]. For instance, mtDNA deletions can result in the loss of critical genes, particularly those encoding respiratory chain proteins, leading to impaired energy metabolism and the progression of metabolic disorders [24-26]. The accumulation of such mutations over time exacerbates mitochondrial dysfunction with age, thereby driving the aging process. This feature has rendered mitochondria a critical model for studying aging and age-related diseases [27, 28]. As research has progressed, it has become increasingly clear that mtDNA mutations may not influence aging in the way initially proposed. Although a temporal association exists between mtDNA mutations and aging, some studies indicate that their accumulation is more likely a secondary consequence of declining energy metabolism rather than a fundamental cause of aging.

In this review, we first outline the causes and dynamics of mtDNA mutations, followed by an exploration of their biological impacts. From the perspective of aging, we summarize and discuss the physiological changes associated with mtDNA mutations, aiming to reconcile the discrepancies between theoretical expectations and empirical findings. Furthermore, we review existing therapeutic strategies and their applicability. Finally, we evaluate the true contribution of mtDNA mutations to the aging process based on current evidence, seeking to elucidate the reasons behind the gap between theoretical models and real-world observations and to bridge this divide.

## Causes of Mutations: mtDNA Replication and Deletion Formation

### mtDNA Replication Process

The accurate expression of mtDNA is fundamental to the biogenesis of the OXPHOS system, ensuring the precision of protein synthesis [29]. The replication of mammalian mtDNA is a process far more intricate and finely regulated than previously assumed. Mammalian mtDNA exists as a small circular double-stranded molecule composed of a heavy strand (H-strand) and a light strand (L-strand), with most of the proteins required for its replication encoded by nDNA. Unlike nDNA replication, mtDNA replication relies on specialized factors, including DNA polymerase γ (Polγ), helicase TWINKLE, and mitochondrial single-stranded DNA-binding protein (mtSSB), among others [30, 31].

Several models have been proposed to describe the mtDNA replication process, with the Strand Displacement Model (SDM) being the earliest and most extensively validated hypothesis [30, 32, 33]. The SDM provides a clear explanation for the formation of the D-loop and the asynchronous replication observed in mtDNA. It has garnered substantial experimental support across mammals and other vertebrates, making it the prevailing model for mtDNA replication. According to this model, replication begins at a specific origin of replication on the heavy strand (OriH), initiating H-strand synthesis. The newly synthesized H-strand displaces the original L-strand, forming a stable three-stranded structure known as the D-loop. Replication of the L-strand only begins when the H-strand synthesis progresses to the second origin of replication, OriL [33, 34]. This temporally segregated replication mechanism stabilizes replication origins and involves the coordinated action of nuclear-encoded proteins, such as Polγ, TWINKLE, mtSSB, and TFAM, in a highly regulated and complex process [35].

A defining feature of the SDM is its stepwise strategy: replication is initiated at OriH to form the D-loop, followed by the subsequent activation of OriL for L-strand replication. This strategy maintains the relative stability of mtDNA by optimizing the balance between regulatory efficiency and simplicity. Yet, the multifunctional nature of the D-loop, along with the inherent features of this mechanism, imposes the trade-off of an elevated mutation rate [32, 33, 36]. Spontaneous errors during replication or faulty repair of damaged DNA bases render mtDNA particularly prone to mutations. These mutations pose a latent threat to the long-term stability of mitochondrial function and are considered significant drivers of various diseases.

### The Fidelity Challenges of mtDNA Replication Mechanisms

Examining the replication mechanisms of mtDNA may help to elucidate the occurrence of deletions and other mutations during the replication process. Research has shown that deletions often arise from replication slippage during the synthesis of the light strand (L-strand) across the major arc.

As described by the model, the preferential replication of the heavy strand (H-strand) in mtDNA exposes the L-strand as single-stranded DNA (ssDNA) for extended periods. These ssDNA regions are highly susceptible to replication fork stalling or slippage, leading to mismatched reannealing or the formation of “palindromic” structures. Specifically, direct repeat sequences within the ssDNA can inadvertently pair, resulting in looped or hairpin structures that extrude portions of the DNA sequence [33, 36]. If these abnormal structures are not corrected during subsequent replication or repair processes, they may be erroneously cleaved or deleted, resulting in deletion mutations. Moreover, during “copy-choice” recombination or the second round of replication, the persistence of such structures can further promote sequence skipping or deletions, exacerbating the accumulation of mutations [36].

Crucially, in the mitochondria’s high oxidative stress environment, ssDNA regions that are exposed or partially exposed for extended periods are particularly vulnerable to oxidative damage. If these lesions are not efficiently repaired, they significantly elevate the risk of point mutations, large-scale deletions, and micro-rearrangements. Consequently, under the Strand Displacement Model (SDM), prolonged exposure of ssDNA regions not only increases the likelihood of replication fork stalling and mismatched reannealing but also facilitates the formation of abnormal secondary structures between direct repeat sequences. These events collectively render mtDNA more prone to mutations and large-scale deletions [37, 38].

On the other hand, although DNA polymerase γ (Polγ) exhibits high DNA synthesis activity, its fidelity is inherently limited [39]. This limitation is particularly pronounced when Polγ operates on single-stranded templates, where its error rate significantly increases. In the absence of accessory subunits, the processivity and fidelity of Polγ decline further [40, 41]. Additionally, due to the relatively weak DNA repair capacity of mitochondria, replication errors introduced by Polγ are less likely to be corrected, creating a permissive environment for mutation accumulation [42].

In parallel, the D-loop region, as a key “triplex” structure in the SDM, exhibits high transcriptional and replicative activity and contains numerous regulatory elements, often identified as a hypervariable region [33, 43, 44]. This region is nonetheless susceptible to transcription-replication conflicts, which elevate the likelihood of DNA breaks or base mismatches, ultimately facilitating the formation of small insertions or deletions [36, 45].

Extensive sequence analyses of mtDNA mutations and mouse model studies have revealed that spontaneous replication errors introduced by Polγ are the primary source of most mtDNA mutations. These findings not only highlight the vulnerabilities of the replication process under the SDM but also underscore the critical roles of Polγ fidelity and mtDNA repair mechanisms in maintaining mtDNA stability [22, 46, 47].

### Inefficient mtDNA Repair Mechanisms

Although mitochondria possess certain DNA repair pathways, their repair capacity is markedly weaker compared to that of nDNA. Errors introduced during the repair process have been identified as a significant source of mtDNA mutations. Among the various repair mechanisms, base excision repair (BER) is the most efficient pathway within mitochondria, effectively addressing oxidative damage in ssDNA lesions [48]. Over and above that, under specific conditions, proteins associated with nucleotide excision repair (NER) can be recruited to mitochondria, where they contribute to safeguarding aging-associated mtDNA from mutational damage [36, 49]. In mice deficient in BER-associated proteins, hepatocytes frequently exhibit abnormal mtDNA rearrangements and deletions [50].

Damage to mtDNA can lead to the loss of affected molecules or result in point mutations, rearrangements, or large-scale deletions. Common sources of damage include replication errors and external harmful stimuli. Recent studies have indicated that, apart from replication slippage, double-strand breaks (DSBs) represent a central mechanism underlying mtDNA deletions [25, 36]. DSBs, which represent a significant threat to nuclear genome stability, physically disrupt chromosomal continuity. In contrast to nDNA, mtDNA preserves its stability primarily by directly degrading damaged molecules rather than relying on conventional repair pathways. Abasic sites or DSBs often initiate the degradation of damaged mtDNA, and given the limited mtDNA copy number, this degradation process frequently stimulates the replication of intact mtDNA to compensate for the loss [51-54]. If linearized mtDNA molecules resulting from DSBs are not swiftly degraded, deletions can occur between free ends or between free ends and regions adjacent to the D-loop [25]. Research indicates that a single DSB in mtDNA, when accompanied by replication fork stalling, can trigger an atypical replication-mediated repair process, ultimately resulting in large-scale mtDNA deletions [55].

Proteins associated with non-homologous end joining (NHEJ), such as XRCC4 and XRCC5, have been identified in mitochondria, but their presence is insufficient to confirm the existence of a complete DSB repair mechanism [56, 57]. Instead, large-scale mtDNA deletions are often found in regions flanked by short direct repeat sequences, suggesting that they may originate from microhomology-mediated end joining (MMEJ), where microhomology sequences are searched and utilized for repair [58, 59]. Not only that, erroneous attempts to repair linearized mtDNA molecules through low-fidelity NHEJ or MMEJ frequently trigger intramolecular recombination events, thereby exacerbating the formation of large deletions [60]. Similar to DSBs, there is currently no definitive evidence supporting the existence of a reliable mismatch repair (MMR) mechanism in mitochondria. In contrast to DSBs, there is also no evidence directly linking MMR to mtDNA mutations.

### Mutations in Replicative Proteins

Replicative proteins are essential molecules for maintaining the integrity and functionality of mtDNA, playing central roles in its replication, repair, and stabilization [52, 61]. Mutations or dysfunctions in these nuclear-encoded proteins often result in mtDNA deletions, depletion, and other pathological changes [62]. Among them, Polγ is the sole DNA polymerase involved in mtDNA replication in humans and is regarded as one of the most critical proteins for mtDNA replication, expression, maintenance, and repair [63]. Mutations in Polγ lead to a broad spectrum of genetic syndromes, including mtDNA mutations, large-scale deletions, multiple deletions, and mtDNA depletion. Studies using mouse models carrying proofreading-deficient mutations in the exonuclease domain of Polγ have shown significant accumulation of large-scale mtDNA deletions [64, 65]. Plus, mutations in the accessory subunit of Polγ (PolγB) are also closely associated with multiple mtDNA deletions [66].

TWINKLE, a mitochondrial 5’-3’ helicase indispensable for mtDNA replication, unwinds double-stranded DNA by disrupting the hydrogen bonds between base pairs, providing single-stranded templates for the replication process [61]. Studies have demonstrated that mutations in TWINKLE can cause replication fork stalling, leading to insufficient mtDNA synthesis and ultimately resulting in mtDNA deletions and depletion [61, 67, 68]. Replication fork stalling induced by mutations in TWINKLE or Polγ is considered a crucial initiating event in the formation of mtDNA deletions.

In addition to Polγ and TWINKLE, other key proteins involved in mtDNA replication—such as SSBP1, PRIMPOL, DNA2, MGME1, and RNASEH1—play essential roles in the replication, synthesis, and processing of the D-loop region [69]. Mutations or functional deficiencies in these proteins frequently destabilize the D-loop, triggering mtDNA rearrangements, deletions, and depletion [70, 71]. These protein defects are directly linked to various mitochondrial diseases, including mtDNA depletion syndrome 11 (MTDPS11) [69]. Collectively, these findings underscore the pivotal role of replicative proteins in maintaining mtDNA stability and mitochondrial function while highlighting their potential pathogenic mechanisms in various mitochondrial disorders.

### Mutations in Transcription Proteins

The interplay between transcription and replication is a fundamental mechanism for maintaining mitochondrial genome stability and regulating mtDNA copy number [69]. This process relies on a critical regulatory network composed of mitochondrial transcription factor A (TFAM), transcription elongation factor (TEFM), transcription factor B2 (TFB2M), and mitochondrial RNA polymerase (POLRMT). TFAM serves a dual function in initiating mtDNA transcription and maintaining genome stability: it directly supports mtDNA replication by promoting RNA primer synthesis and regulates the packaging and unwinding of mtDNA to ensure efficient replication initiation [46, 72]. TEFM plays a pivotal role in transcription elongation, preventing premature termination of long-chain RNA synthesis, which ensures the production of sufficient quantities of RNA primers critical for replication efficiency [62, 73, 74]. TFB2M, through direct interaction with POLRMT, activates transcription initiation and further reinforces the tight coupling between transcription and replication [75, 76].

Notably, recent studies have shown that any disruption in the transcription complex, including functional loss or regulatory abnormalities in these factors, poses a potential threat to mtDNA stability. These findings underscore the essential role of transcription-replication coordination in maintaining mitochondrial genome integrity and highlight the necessity of understanding the functions of the transcription complex in mtDNA stability regulation. Such insights are vital for elucidating the mechanisms underlying mitochondrial-associated diseases and developing effective therapeutic strategies [52, 74, 77].

## mtDNA Mutations: Controversies from the Perspective of Aging

### Energy Metabolism

As the central source of cellular energy, mitochondrial dysfunction has long been considered a key driver of aging [8, 78]. With advancing age, mtDNA mutations accumulate progressively and are closely associated with the aging process [79]. Across various aging models, OXPHOS dysfunction is commonly identified as a critical link between aging and energy metabolism dysregulation [80]. The 13 proteins encoded by mtDNA are integral components of the mitochondrial respiratory chain complexes, directly involved in electron transport. Consequently, mtDNA mutations lead to impaired energy metabolism, which drives the aging process. Some hypotheses suggest that mutated mtDNA may have a replication advantage over wild-type mtDNA, indirectly supporting their accumulation [81-83]. The build-up of deleted mtDNA results in respiratory chain dysfunction, manifesting as premature aging phenotypes and shortened lifespan [65, 84, 85]. As well, significant mtDNA depletion has been observed in age-related neurodegenerative diseases such as Alzheimer’s and Parkinson’s diseases [86-88].

Interestingly, complete depletion or substantial reduction of mtDNA content (as seen in ρ0 cells) has been shown, under specific conditions, to alleviate aging phenotypes associated with OXPHOS dysfunction. In some cases, it even inhibits or reverses markers of cellular senescence [89]. This phenomenon is often described as suppressing or partially reversing cellular aging markers. These observations seem to support the hypothesis that mtDNA mutations are pivotal in aging, though this conclusion is contested by findings indicating that certain mtDNA mutations, despite causing phenotypic changes, exert minimal or no influence on lifespan [90, 91].

The primary way mtDNA mutations contribute to aging is by impairing OXPHOS, thereby accelerating the process. The debate, however, centers on whether the mtDNA mutations that accumulate during normal aging are substantial enough to significantly compromise ATP production. Some studies propose that the impact of these mutations is generally mild and unlikely to cause widespread aging or dysfunction [79, 80]. In normal aging, mtDNA mutations rarely reach levels that extensively impair OXPHOS function, nor do all cells accumulate high levels of mutations. Even in cells with significant mutation loads, their functional deficits may be compensated for by neighboring cells or tissues. One possible explanation is that the lack of a pronounced “mutation-dysfunction” effect may be attributed to the mitochondrial threshold effect, wherein pathogenic mutations must exceed 70%-90% of mtDNA copies to meaningfully impact mitochondrial function. Even when dysfunction occurs, mitochondrial quality control mechanisms can partially eliminate damaged mitochondria, mitigating their effects.

The accumulation of mtDNA mutations increases with age and exhibits notable tissue specificity. Focusing on tissues with higher mutation burdens and integrating these findings with longitudinal studies on healthy aging could reveal novel biological patterns [92-94]. Further investigation into the relationship between “aging-mutation-phenotype” requires a focus on specific tissues or cells, comparing healthy aging to localized pathological aging to elucidate the true contribution of mtDNA mutations.

Recent studies have highlighted an intriguing connection: OXPHOS defects caused by age-dependent mtDNA mutations may upregulate the serine biosynthesis pathway, inducing metabolic reprogramming and promoting the rapid growth and survival of tumor cells [95]. This suggests that mtDNA mutations are not only linked to aging but may also facilitate cancer cell growth through anabolic remodeling. Beyond that, cellular senescence is often regarded as a tumor-suppressive mechanism, while tumorigenesis represents the escape of cells from the constraints of senescence. In this delicate “aging-tumor” balance, mtDNA mutations exhibit a complex bidirectional role [96]. For instance, the same random mutation may have distinct effects depending on the cellular context, offering paradoxical insights into reversing aging or promoting cancer cell senescence.

### Oxidative Stress

Although mtDNA mutations do not show a direct causal relationship with the aging process, cells consistently accumulate mtDNA mutations throughout life. Hypotheses such as the reductive hotspot hypothesis and the activation of the NLRP3 inflammasome suggest that mtDNA mutations induce cellular toxicity, affecting surrounding cells. This toxicity arises from OXPHOS impairment and superoxide production by NLRP3, impacting the immune system and influencing other hallmarks of aging [79, 97]. Based on these observations, oxidative stress has long been considered a key driver of aging. Albeit, the free radical theory of aging has increasingly faced criticism.

Oxidative stress remains a focal event in aging, capable of acting as both a cause and a consequence. This “jack of all trades” hypothesis made the free radical theory of aging an initially popular framework. The theory posits that mtDNA mutations impair OXPHOS, leading to increased ROS production. Excess ROS not only damages mtDNA but also depletes cellular reducing agents and disrupts antioxidant defense systems. Ultimately, this cascade results in DNA damage, including mtDNA, as well as the degradation of proteins and lipids, forming a vicious cycle. This seemingly intuitive hypothesis has gained partial support, as it has been observed in various pathological processes, including ischemia and hypoxia in neuronal cells [98, 99].

Much like the mtDNA mutation hypothesis, the free radical theory has encountered substantial challenges under closer scrutiny. The proposed vicious cycle has been notoriously difficult to confirm through experimental validation, underscoring the disconnect between theoretical models and empirical findings. A notable example lies in the observation that while reductions in antioxidant enzyme levels can elevate mtDNA damage, they appear to have little to no impact on lifespan [92, 100]. Similarly, supplementation with antioxidants fails to eliminate senescent cells or extend lifespan and may even be harmful, potentially shortening lifespan in certain cases [101-103].

Conversely, some experimental results suggest that moderate levels of oxidative stress may enhance cellular survival and longevity [104]. The mitohormesis hypothesis proposes that mild mitochondrial stress can reprogram cellular metabolism, protein-folding capacity, and the redox environment, rendering cells or organisms more resilient to future stress [105, 106]. ROS serve dual roles in cells: on one hand, they act as drivers of DNA damage, while on the other, they function as second messengers in cellular signal transduction [107, 108]. Excessive antioxidant supplementation might interfere with normal cellular stress responses, weakening adaptive and defensive mechanisms [105, 109]. Moderate exercise, for example, can enhance antioxidant defenses and resilience by stimulating antioxidant enzyme activity and efficiency, thereby reducing mtDNA mutation burden and mitigating premature aging [110-112].

A reasonable hypothesis is that increased ROS levels are more likely a consequence of senescence rather than its cause. ROS may act as signaling molecules, mediating downstream responses to cellular damage. Current evidence increasingly suggests that free radicals and oxidative damage are not primary drivers of aging and may not even be the main contributors to mtDNA mutations. The primary source of mtDNA mutations is still believed to be replication errors, rather than oxidative damage [8, 23, 113, 114]. While ROS contribute to oxidative DNA base damage products, such as 8-oxoG, the frequency of 8-oxoG formation is much lower than that of random mtDNA mutations. For instance, although ogg1 or ogg1/mth1 knockout mice show elevated 8-oxoG levels in mtDNA, the overall mutation frequency in mtDNA does not significantly increase [115, 116]. Oxidative cytosine damage primarily leads to G→T/C→A transversions, while G→A/C→T transitions are the most common mutations caused by oxidative DNA damage [117]. Numerous studies indicate that these oxidative mutations are not major contributors to mtDNA mutagenesis and do not significantly increase with age. For example, in aging mouse livers, such mutations account for only 12% of total mutations, whereas replication error-related mutations constitute 68% [93, 114, 118-121].

The relationship between ROS levels, aging, lifespan, and health is not a simple linear one, nor is oxidative stress a unidirectional accumulation. Aging results from the interplay of multiple factors—a synergistic accumulation of diverse damage. Simple, direct hypotheses cannot fully explain this complex process. Applying the free radical theory to aging is akin to using Newtonian mechanics to explain quantum entanglement: it captures part of the truth but overlooks deeper underlying logic. Similar to the hypotheses mentioned earlier, rather than emphasizing the minimal impact of ROS damage on mtDNA, it is more plausible that mechanisms for the recognition and removal of ROS-induced damage or mutations maintain a steady state during aging [93]. Compared to antioxidants, more proactive mitochondrial quality control or repair strategies appear to be more effective options [122]. For instance, mitochondria with high ROS levels that damage their genomes may become targets for mitophagy or face replication inhibition [123]. This indirectly prevents the spread of dangerous signals. Mitophagy, as part of mitochondrial quality control, reduces mtDNA leakage and subsequent cGAS/STING-dependent inflammatory responses, promoting healthy aging [124].

Cells, like organisms, do not passively endure ROS but respond adaptively depending on the context. Similarly, aging research has made tremendous strides in addressing external factors and understanding the process. The free radical theory, though initially appealing, is an oversimplified model that requires refinement as research progresses [113]. Employing more precise detection techniques, broader observations, and integrating multiple hypotheses are essential to comprehensively understand the interconnected factors driving aging, rather than isolating individual phenotypes in the process.

### Apoptosis

As a programmed cell death process, apoptosis removes damaged, senescent, or excessive cells, maintaining growth, tissue homeostasis, and immune system balance while preventing abnormal cells from causing pathological damage. It serves as a crucial quality control mechanism. Earlier theories and studies suggested that aging promotes increased apoptosis, leading to damage. In contrast, more extensive recent research reveals that senescent cells typically display anti-apoptotic characteristics [125]. At the cellular level, apoptosis and senescence represent two distinct fates for damaged cells, but senescent cells often display reduced sensitivity to apoptotic signals due to upregulation of anti-apoptotic proteins such as Bcl-2 and inhibitors of apoptosis proteins (IAPs) through various pathways. Altered activity of p21, p16, and p53 further drives cells into long-term cell cycle arrest, rendering them more resistant to apoptosis [126].

This resistance to apoptosis helps maintain tissue homeostasis to some extent but simultaneously increases the burden of senescent cells on their microenvironment [127]. Promoting apoptosis has been shown to effectively eliminate senescent cells, with mtDNA playing a role in facilitating this process [128, 129]. For example, high doses of ganciclovir (GCV) combined with herpes simplex virus thymidine kinase (HSVtk) can initiate caspase-dependent apoptosis by increasing mtDNA mutation rates and damage, effectively targeting and eradicating senescent cells [130]. Similarly, the use of siRNAs or drugs to inhibit BCL-family proteins has emerged as another effective approach to induce apoptosis specifically in senescent cells, reducing their accumulation [131, 132].

The intrinsic apoptotic pathway becomes upregulated as mtDNA mutations accumulate, accompanied by the suppression of anti-apoptotic proteins. This shift is characterized by increased Bax expression and decreased Bcl-2 levels [133, 134]. Altered BAX and BAK expressions facilitate macropore formation on the mitochondrial outer membrane, enhancing its permeability. Consequently, cytochrome c is released into the cytosol, activating caspases and initiating the execution phase of apoptosis [135, 136]. At the same time, mtDNA leakage occurs, activating the cGAS/STING pathway and promoting the secretion of the senescence-associated secretory phenotype (SASP). This cascade drives chronic inflammation, induces tissue damage, and ultimately shortens healthspan [137, 138].Overall, while promoting apoptosis offers a strategy to eliminate senescent cells, the associated side effects, including inflammation and tissue damage, may outweigh the benefits. A more measured approach involving controlled levels of apoptosis might strike a balance, reducing the detrimental effects of senescent cells on surrounding tissues without causing excessive harm.

### MitoEpigenetics

Mitoepigenetics, a burgeoning field of research, focuses on the epigenetic modifications of mtDNA and their roles in biological processes. Similar to nuclear epigenetics, these modifications do not involve changes in the DNA sequence itself [139]. While mitoepigenetic modifications appear to be more limited compared to those of nDNA, they play critical roles in regulating mitochondrial function and influencing cellular states [140]. Changes in mitoepigenetic markers affect a range of biological processes, including cell cycle regulation, responses to environmental stress, mitochondrial physiological activity, and OXPHOS functionality. What is more to environmental and physiological factors, mtDNA mutations are significant drivers of epigenetic reprogramming in both mitochondria and the nucleus [141].

The primary epigenetic modifications in mtDNA include methylation, hydroxymethylation, and acetylation [142, 143]. While these changes help cells and organisms adapt to environmental stimuli, they may also introduce adverse effects, potentially accelerating aging [144]. Proper DNMT1 expression and activity are prerequisites for maintaining epigenetic stability and play a vital role in regulating mtDNA expression [142]. The activity of mtDNMT1 is altered by mtDNA mutations, directly disrupting the methylation patterns of mtDNA [145, 146]. Aberrant methylation patterns may result in abnormal mitochondrial gene expression [147]. Studies have shown that mtDNA mutations in oocytes are associated with significant epigenetic changes, negatively impacting embryonic development [147, 148]. These mutations may lead to reduced global DNA methylation in oocytes, insufficient zygotic genome demethylation, and dysregulated gene expression [149]. Furthermore, decreased mtDNA methylation levels have been linked to aging through mechanisms such as increased inflammation [139].

Changes in mitoepigenetics are pivotal for the crosstalk between the nuclear and mitochondrial genomes. Certain mtDNA alterations correlate with changes in nDNA methylation levels, and nDNA encodes many essential proteins required for mtDNA function [150-152]. For instance, Polγ, a protein encoded by nuclear genes, has its methylation status linked to the regulation of mtDNA copy number [153]. mtDNA copy number is a critical indicator of mitochondrial functionality and a key factor in resisting mutations and damage. Its regulation is also influenced by changes in mtDNA methylation [152, 154].

Research on mitoepigenetic remains less extensive than that on nuclear epigenetics, and many hypotheses require further investigation. One area of debate is how mitochondrial double membranes restrict nuclear DNMTs from accessing mtDNA. Classical DNMTs—DNMT1, DNMT3a, and DNMT3b—are well-established in nDNA methylation, but only DNMT1 has been implicated in mtDNA methylation, potentially relying on specific localization signals [142, 155]. Even so, the role of DNMT1 in mtDNA methylation is not yet fully understood, leaving open the possibility of “non-classical methylation” processes.

Hydroxymethylation, another important epigenetic modification, also raises questions about mitoepigenetic remodeling. Unlike nDNA, where the interconversion of 5mC and 5hmC depends on TET enzymes, such enzymes have not been identified in mitochondria. This suggests that mitoepigenetic remodeling may rely on alternative pathways [156]. Despite these uncertainties, the existence of mitoepigenetic is well-supported. Recent studies have shown that the loss of epigenetic information disrupts a cell’s ability to maintain its identity, contributing to aging in mammals [157]. Epigenetic changes within mitochondria may similarly influence the aging process. The informational theory of aging also supports the notion that mitochondrial epigenetics gradually evolve over time. Such changes, though, are unlikely to compromise mitochondrial identity.

To quantify epigenetic aging, various epigenetic clocks have been developed to predict the rate of aging. These clocks provide insights into an individual’s overall aging trajectory and mortality risk [158]. Similar to nuclear epigenetic clocks, somatic mitoepigenetic changes and the accumulation of mutations exhibit clock-like behavior [93, 98, 119, 159, 160]. These epigenetic changes and mutation accumulations hold promise as potential tools for predicting aging, offering novel insights into the mechanisms underlying the aging process.

### mtDNA Mutations: Tentatively Accepted Processes

In addition to the controversial hypotheses discussed earlier, mtDNA mutations undeniably contribute to the aging process in several ways. One hallmark of aging is the permanent arrest of the cell cycle, with mtDNA damage serving as an upstream signal that triggers cell cycle arrest in mammalian cells [161, 162]. The G1/S and G2/M checkpoints act as surveillance mechanisms, halting the cell cycle to prevent the propagation of damaged DNA [163]. The mitochondrial metabolic checkpoint monitors mitochondrial health, responding to stress signals from mtDNA mutations [164]. DNA damage responses, such as DSBs induced by mtDNA mutations, activate these checkpoints. Through mitochondria-to-nucleus retrograde signaling, these responses alter p53/p21 activity, inducing cell cycle arrest to prevent the dissemination of harmful genetic material [165-168].

While cell cycle arrest allows time for DNA repair, aging significantly increases the burden on both mtDNA and nDNA repair mechanisms while simultaneously impairing their efficiency. This decline in repair capacity results in cells failing to recover and entering a state of permanent arrest [169-171].

Another critical impact of mtDNA mutations on aging is their influence on nDNA [27, 172]. Given the communication between mtDNA and nDNA, extensive mtDNA mutations can compromise nuclear genome stability, leading to slowed DNA replication and the occurrence of DSBs in proliferating cells [145, 147]. Beyond that, mtDNA fragments can integrate into nDNA, disrupting normal gene expression and causing epigenetic dysregulation, further compromising lifespan and cellular function [161, 173].

### mtDNA Mutations as Targets to Mitigate Aging

Intervening in mtDNA mutations has become a focal area in efforts to delay aging. Therapeutics such as CoQ10 and nicotinamide mononucleotide (NMN) have demonstrated efficacy in populations suffering from neurodegenerative and cardiovascular diseases [174, 175]. Autophagy offers an effective mechanism for removing mutated mtDNA, with moderate activation preventing the accumulation of such mutations [11, 176]. By stimulating autophagy, activating the PINK1/Parkin pathway, or selectively reducing mitochondrial protein levels, mutated mtDNA can be efficiently diminished [79, 177]. The use of small-molecule modulators to enhance mitophagy has emerged as a promising strategy for treating mitochondrial damage-related diseases, including Parkinson’s disease [123, 178, 179]. Looking ahead, precision-targeting technologies are poised to transform therapeutic approaches in this domain.

An effective method to reduce mtDNA heteroplasmy involves 5S rRNA molecules, although challenges remain in improving their cellular expression efficiency [180]. Novel peptide/pDNA complexes incorporating mitochondrial-targeting sequences have proven effective in optimizing mitochondrial gene expression [181]. Over and above that, liposomal systems such as rRNA-MITO-Porter, which deliver encapsulated therapeutic rRNA into mitochondria via membrane fusion, have shown promise in enhancing mitochondrial function [182].

When mitochondrial heteroplasmy surpasses the threshold of functional capacity, it becomes a primary driver of dysfunction. Reducing heteroplasmy is therefore critical to improving mitochondrial health. Advances in gene-editing technologies now enable precise editing of mtDNA [183]. Tools such as TALENs and zinc finger nucleases (ZFNs) show significant potential in selectively eliminating or replacing mutated mtDNA [184]. For instance, heterodimeric mtZFN constructs have demonstrated moderate success in shifting heteroplasmy in cybrid cell models carrying mutations such as m.8993T>G or common deletions, thereby restoring mitochondrial function [185]. Refinements in these tools have achieved near-complete elimination of mutated mtDNA in certain experiments [186, 187]. Similarly, mitoTALENs and their variants have been utilized to manipulate heteroplasmy in induced pluripotent stem cells (iPSCs) with mutations such as m.3243A>G and m.13513G>A, as well as in oocytes through cytoplasmic transfer [188, 189]. Challenges related to the intrinsic specificity of the TevI nuclease domain and linker sequences, as well as difficulties associated with constitutively active nucleases, may limit further applications [190]. Nonetheless, TALENs and ZFNs achieve site-specific cleavage, effectively degrading mutated mtDNA and reducing mutation load. In vivo studies have validated these tools in restoring respiratory function and energy metabolism in affected tissues [187, 191].

While programmable tools for shifting heteroplasmy offer the potential to nearly eliminate mtDNA heterogeneity, they face certain limitations, such as their large molecular size, heterodimeric structure, and limited flexibility in targeting single base changes. Mitochondrial-targeted nucleases based on I-CreI (mitoARCUS) address these issues, demonstrating robust elimination of mutated mtDNA in liver and skeletal muscle, alongside restored MT-tRNA^Ala^ levels [192].

Compared to conventional antioxidants or energy supplements, these cutting-edge approaches provide greater precision and longer-lasting benefits in reducing mtDNA mutation burdens[193]. The findings from these preclinical studies are compelling, yet certain challenges have prevented their transition from preclinical research to clinical application [194, 195]. Safety is the foremost concern, particularly regarding potential off-target effects. Although mtZFNs have shown no evidence of off-target activity in nDNA, mitoTALENs are still under investigation [186, 191]. Given their mitochondrial localization, any off-target effects would likely result in double-strand break-induced degradation rather than the generation of new mutations [59, 196]. Nevertheless, rigorous testing is required to confirm the safety of these tools in complex biological systems. Another major limitation is delivery capacity. The scalability and safety of recombinant AAV delivery systems remain challenging, as large doses required for adult applications could compromise safety. Moreover, the immunogenicity of bacterial-derived FokI catalytic domains and TALE DNA-binding domains within AAV vectors has yet to be fully addressed [197].

The advent of DddA-derived cytosine base editors (DdCBEs), which catalyze C→T (G→A) transitions by deaminating cytosines in TC motifs on double-stranded DNA, has overcome some limitations of earlier systems that targeted only single-stranded DNA [198]. DdCBEs have been refined to enhance specificity, with HiFi-DdCBE significantly reducing off-target binding [199-202]. Zinc finger deaminases (ZFDs), which utilize DdCBEs with zinc finger targeting proteins, achieve approximately 30% mtDNA base editing efficiency while avoiding unwanted bystander editing [203]. Another notable advancement is the development of TALE-linked deaminases (TALEDs), which enable A-to-G base conversions in mtDNA [204]. The ability to modify A·T base pairs into G·C without sequence context constraints significantly expands the range of disease-associated mtDNA mutations that can be targeted, addressing the limitations of DdCBEs and ZFDs, which are restricted to editing TC motifs. A remaining challenge is the precise localization of A or T within the editing window [202]. Monomeric DdCBEs (mDdCBEs), with their smaller size and requirement for only one TALE-binding site, enhance flexibility and address delivery issues by being compatible with AAV packaging [205].

These technological advancements have revolutionized mtDNA editing, significantly contributing to the reduction of mtDNA heterogeneity. While these tools hold immense potential for mitigating aging through heteroplasmy reduction, the gap between laboratory innovation and clinical application remains substantial. Aging, as underscored by the lessons of the free radical theory, is a complex, multifaceted process that cannot be fully addressed by single interventions. The inherent heterogeneity and complexity of aging make it challenging to discern the precise contribution of mtDNA mutations to disease pathophysiology. Currently, mtDNA editing is fundamentally limited to targeting specific types of mutations, restricted to purine-to-pyrimidine or pyrimidine-to-purine point mutations. Modifications involving insertions, deletions, or multi-nucleotide changes remain unattainable [202].

While traditional energy supplements continue to play a role in mitigating mitochondrial dysfunction, next-generation mtDNA-targeted gene-editing technologies offer unparalleled precision and therapeutic potential. Before mtDNA base-editing technologies can be translated into human applications, further safety studies are essential. Advancing more robust editing techniques and facilitating their translation will be critical steps toward unlocking the potential of mitochondrial genome therapies.

## mtDNA Mutations in Aging Represent the Vector Resulting from Multiple Combined Factors

Mitochondria and their DNA have garnered significant attention due to their crucial role in cellular and organismal physiology. mtDNA, characterized by its high mutation rate and error-prone replication process owing to limited proofreading mechanisms, has traditionally been considered a major contributor to various pathological changes. Given the strong age-related accumulation of mtDNA mutations, many theories have proposed these mutations as central drivers of aging. Within the comprehensive theoretical framework of aging, energy metabolism and oxidative stress, intricately linked to mitochondrial function, have garnered significant attention. Intuitive hypotheses, such as the free radical theory of aging, have encountered substantial challenges posed by emerging research findings. Rather than being solely the accumulation of linear damage, aging arises from the complex interplay of metabolism, signaling pathways, genetic factors, and environmental influences. Free radicals, while central to some theories, are merely one component of a broader network, with their physiological functions being far more diverse and context-dependent than previously assumed. These seemingly contradictory realities do not necessarily invalidate existing theories but rather highlight the overly idealized nature of their models.

The role of mtDNA mutations in aging and lifespan remains multifaceted. Current evidence suggests that the contribution of mtDNA mutations to aging and lifespan may have been overestimated in the past [113, 206, 207]. While mtDNA mutations undoubtedly promote aging and certain premature aging phenotypes through multiple pathways, their overall impact on organismal physiology is less pronounced than previously believed [207, 208]. Within the multifactorial regulatory network of aging, mtDNA mutations represent just one of many influences rather than a singular determinant. Moreover, cells possess robust repair and defense mechanisms, with mtDNA mutations actively stimulating these protective responses. On a macroscopic level, observed outcomes such as lifespan are shaped by the cumulative effects of numerous factors, which obscure the contributions of individual components. As a result, it is difficult to definitively validate or refute existing theories.

Beyond individual variability, species specificity and environmental factors significantly influence the relationship between mtDNA mutations, aging, and lifespan. In species like Caenorhabditis elegans and Drosophila, the accumulation of mtDNA mutations shows no clear association with shortened lifespan [92]. These organisms may possess mechanisms to mitigate the negative effects of mtDNA mutations [209]. Interestingly, not all mtDNA mutations negatively impact aging. In certain cases, mtDNA mutations that increase ROS levels or mildly impair mitochondrial function have been shown to extend lifespan in mice and nematodes, while antioxidant interventions negated this lifespan extension in mice [210, 211]. In mammals and humans, this relationship is far more complex, encompassing regulatory mechanisms and compensatory phenomena [91, 212].

The current understanding of mtDNA mutations can be partially explained by several hypotheses. Mitochondria, being maternally inherited and lacking the homologous recombination afforded by sexual reproduction, allow mtDNA mutations to accumulate across generations. While many mutations may impair mitochondrial function, mechanisms such as the “bottleneck effect” and “threshold effect” enable cells to tolerate a certain level of mutations, maintaining essential mitochondrial functions while preserving potential genetic diversity and adaptability [213-217]. According to the “Red Queen Hypothesis,” organisms must continuously evolve to survive in a dynamic environment. Similarly, mitochondria, despite lacking traditional sexual recombination, achieve genetic renewal through a combination of high mutation rates and repair or clearance systems. This allows mitochondria to adapt their genomes to changes in both the host and external environment [218, 219]. Favorable mutations that arise sporadically may be enriched across generations, providing survival or reproductive advantages. This represents a micro-level process of “self-evolution” wherein most mutations are deleterious but occasionally yield beneficial variations that drive mitochondrial and host evolution [220, 221]. Even so, these conclusions remain constrained, relying largely on numerous inferences and relatively limited evidence.

These seemingly contradictory findings can be distilled into a simple model: the interplay between pro-aging and anti-aging forces. The vector sum of these forces ultimately determines an organism’s fate. Be that as it may, many factors are not binary. mtDNA mutations exemplify this complexity. While they may activate repair and defense mechanisms and their negative effects (e.g., on nDNA stability, cell cycle arrest, or inflammation) are evident, their overall impact is moderated by other interacting variables. On the organismal or population level, heterogeneity either dilutes or amplifies their influence, leading to vastly different conclusions depending on the perspective taken.

Current research is limited by methodological constraints, and interpreting the functional roles of random mutations requires a multi-layered, spatiotemporal approach to fully grasp their implications. Integrating multiple hypotheses with advanced analytical tools to investigate mtDNA mutation patterns across diverse cell types and disease states will be essential. Collaborative analyses incorporating other influencing factors are needed to achieve a comprehensive understanding[222]. Only by adopting a higher-dimensional perspective can we simplify and deconstruct this intricate process, uncovering the true nature of mtDNA mutations in aging.
